# A Potential New Source of Therapeutic Agents for the Treatment of Mucocutaneous Leishmaniasis: The Essential Oil of *Rhaphiodon echinus*

**DOI:** 10.3390/molecules27072169

**Published:** 2022-03-27

**Authors:** Carlos Vinicius Barros Oliveira, Patric Anderson Gomes da Silva, Saulo Relison Tintino, Cathia Cecília Coronel, Maria Celeste Vega Gomez, Mírian Rolón, Francisco Assis Bezerra da Cunha, Maria Flaviana Bezerra Morais-Braga, Henrique Douglas Melo Coutinho, Abolghasem Siyadatpanah, Polrat Wilairatana, Jean Paul Kamdem, Luiz Marivando Barros, Antonia Eliene Duarte, Pedro Silvino Pereira

**Affiliations:** 1 Microscopy Laboratory, Regional University of Cariri (URCA), 1161 Cel. Antonio Luiz Avenue, Crato 63105-000, CE, Brazil; viniciusbluesky@gmail.com (C.V.B.O.); patricanderson16@icloud.com (P.A.G.d.S.); kamdemjeanpaul2005@yahoo.fr (J.P.K.); marivando.barros@urca.br (L.M.B.); duarte105@yahoo.com.br (A.E.D.); 2 Laboratory of Microbiology and Molecular Biology, Regional University of Cariri (URCA), 1161 Cel. Antonio Luiz Avenue, Crato 63105-000, CE, Brazil; saulorelison@gmail.com (S.R.T.); cunha.urca@gmail.com (F.A.B.d.C.); flavianamoraisb@yahoo.com.br (M.F.B.M.-B.); 3 Centro Para El Desarrollo De La Investigación Científica (CEDIC), Fundación Moisés Bertoni/Laboratorios Dıáz Gill, Manduvira 635, Asunción CP. 1255, Paraguay; cathiacoronel@gmail.com (C.C.C.); mcvegagomez@gmail.com (M.C.V.G.); rolonmiriam@gmail.com (M.R.); 4 Ferdows School of Paramedical and Health, Birjand University of Medical Sciences, Birjand 9717853577, Iran; 5 Department of Clinical Tropical Medicine, Faculty of Tropical Medicine, Mahidol University, Bangkok 10400, Thailand

**Keywords:** trypanocidal, leishmanicidal, *Rhaphiodon echinus*, GC–MS

## Abstract

Weeds are an important source of natural products; with promising biological activity. This study investigated the anti-kinetoplastida potential (in vitro) to evaluate the cytotoxicity (in vitro) and antioxidant capacity of the essential oil of *Rhaphiodon echinus* (EORe), which is an infesting plant species. The essential oil was analyzed by GC/MS. The antioxidant capacity was evaluated by reduction of the DPPH radical and Fe^3+^ ion. The clone *Trypanosoma cruzi* CL-B5 was used to search for anti-epimastigote activity. Antileishmanial activity was determined using promastigotes of *Leishmania braziliensis* (MHOM/CW/88/UA301). NCTC 929 fibroblasts were used for the cytotoxicity test. The results showed that the main constituent of the essential oil was γ-elemene. No relevant effect was observed concerning the ability to reduce the DPPH radical; only at the concentration of 480 μg/mL did the essential oil demonstrate a high reduction of Fe^3+^ power. The oil was active against *L. brasiliensis* promastigotes; but not against the epimastigote form of *T. cruzi*. Cytotoxicity for mammalian cells was low at the active concentration capable of killing more than 70% of promastigote forms. The results revealed that the essential oil of *R. echinus* showed activity against *L. brasiliensis*; positioning itself as a promising agent for antileishmanial therapies.

## 1. Introduction

Protozoan parasites of the *Leishmania* genus causes a disease, known collectively as leishmaniasis, which affects numerous body systems (e.g., the epithelial and the muscular). About 1.3 million new cases and approximately 20,000 to 30,000 deaths from this pathology are reported annually. The disease is highly endemic in the Indian subcontinent and in East Africa, and more than 90% of cases occur in Bangladesh, Brazil, Ethiopia, India, South Sudan, and Sudan [1]. The Leishmania genus includes several species known to cause diseases in humans, so that, among cold- and warm-blooded vertebrates, they are transmitted by insects of the genera *Phlebotomus* and *Lutzomyia* [2].

Miltefosine (Impavido©), in a modern therapeutic scenario against this pathogen, is a new drug with proven efficacy in the treatment of visceral and cutaneous leishmaniasis, including cases of mucocutaneous leishmaniasis by *L. brasiliensis* [3]. However, there are many factors that prevent its use, e.g., administration difficulty, duration of treatment, toxicity, the development of parasitic resistance to drugs, and the lack of scientific evidence on efficacy, indicate the need for alternative therapies [4].

Chagas disease is a major public health problem in developing countries in Latin America, affecting the poor and rural populations [4]. It is caused by the protozoa *Trypanosoma cruzi* (*T. cruzi*) and is primarily transmitted by domestic and sylvatic insects of the subfamily *Triatominae*; it may be also transmitted to humans congenitally, by blood transfusion, organ transplants, and by the oral route [5]. Approximately 6 to 7 million people worldwide are estimated to be infected with *Trypanosoma cruzi*; the disease is primarily found in endemic areas of 21 continental Latin American countries [6].

Given this context, the identification of new, safe, and more effective therapeutic agents with antiparasitic action is desirable, and the plants are considered a promising sources of active phytochemicals [7]. The weed flora is an important part of biodiversity that has shown different types of activities, such as antimicrobial [8], anti-inflammatory, antinociceptive, anti-hyperglycemic [9], among others.

The species *Rhaphiodon echinus* (Nees and Mart.) Schauer (synonyms: *Hyptis sideritis* Mart ex. Benth; *Zappania echinus* Nees and Mart.; *Lippia echinus* (Nees and Mart.) Spreng.; *Mesosphaerum sideritis* (Mart. ex Benth.) Kuntze) [10], is a plant of the family *Lamiaceae* found in various environments, such as vacant lots and caatinga area. Research indicates that this plant has pharmacological potential; it is shown to be active in antimicrobial activity [11], anti-inflammatory, analgesic activity [12], and antioxidant assays, evidenced by the activity against the DPPH radical in a concentration-dependent manner [13].

The practice of producing and analyzing bibliometric networks, often referred to as ‘scientific mapping’, has received much attention since the beginnings of bibliometric research, since it makes it possible to determine trends in scientific production related to a given topic and even indicate strong correlations, such as those between natural products and their main applications [14]. Visualization analyses are efficiently used to study a wide variety of bibliometric networks, from networks of citation relationships between two publications or journals to networks of co-authorship relationships between researchers, or networks of co-occurrence relationships between keywords [15].

In this study, with the intention of exploring a possible new natural source of therapeutic agents for the treatment of mucocutaneous leishmaniasis and/or Chagas disease, we investigated the anti-kinetoplastida potential, as well as the cytotoxicity and antioxidant capacities of the essential oil of *Rhaphiodon echinus* (EORe), which is an infesting plant species.

## 2. Results

### 2.1. Essential Oil Obtained and Chemical Analysis

The essential oil of the dried leaves of *R. echinus* had a yield of 0.12%. A total of 21 compounds were identified from the chemical analysis of the essential oil, where most of the identified compounds belong to the sesquiterpenes class. Their retention indices and relative quantity, listed in order of elution, are shown in Table 1.

The analyses showed that γ-elemene, a hydrocarbon sesquiterpene, was predominant in essential oil, counting for more than 21.83% of its composition. Moreover, α-bisabolene (12.82%), caryophyllene oxide (10.61%), and germacrene D (10.30%) were also representative, with concentrations higher than 10%.

### 2.2. Visualization Analysis of Descriptor Networks

**Figure 1:**
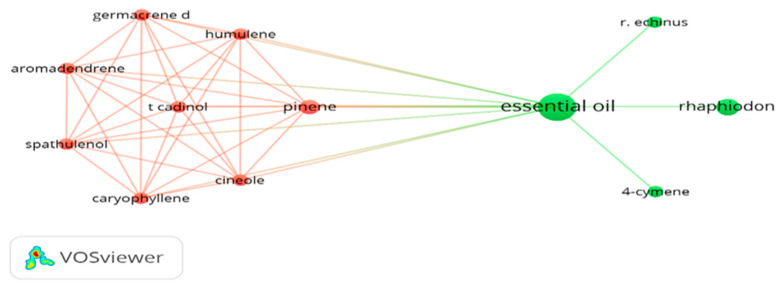
Co-occurrence analysis of components obtained in the essential oil of *Rhaphiodon echinus*. Note: the size of the circle or node is equivalent to the occurrence number of the descriptor.

The software found 583 possible keywords, so it defined at least one occurrence as a minimum limit. However, only the components obtained in the essential oil were selected for the creation of the visualization network. In this case, the terms selected were: aromadendrene, caryophyllene oxide, cineole, copaene, germacrene D, spathulenol, α-humulene, β-cymene, β-pinene, δ-cadinol, “essential oil”, *Rhaphiodon*, and “*R. echinus*”. As shown in Figure 1, the system identified two clusters; we observed that, although each cluster complements the connections, they are very limited, in this case, it is a subject that still needs to be explored by the scientific community.

### 2.3. Effect of the Essential Oil on DPPH Radicals

**Figure 2:**
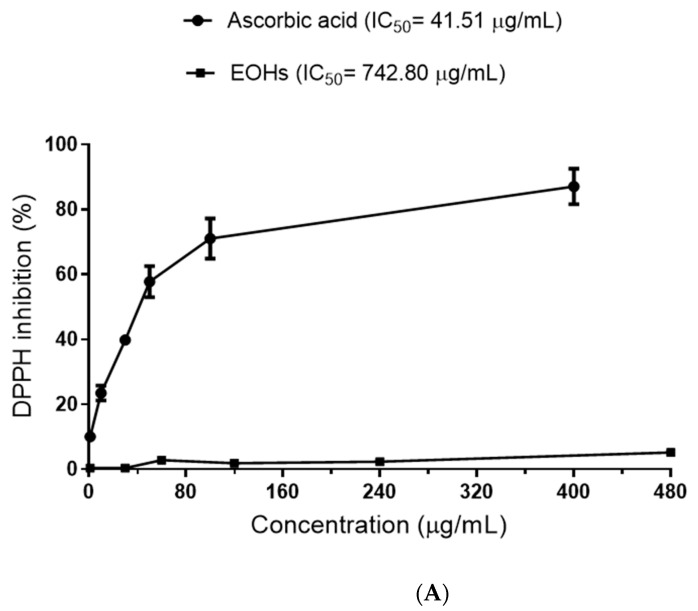

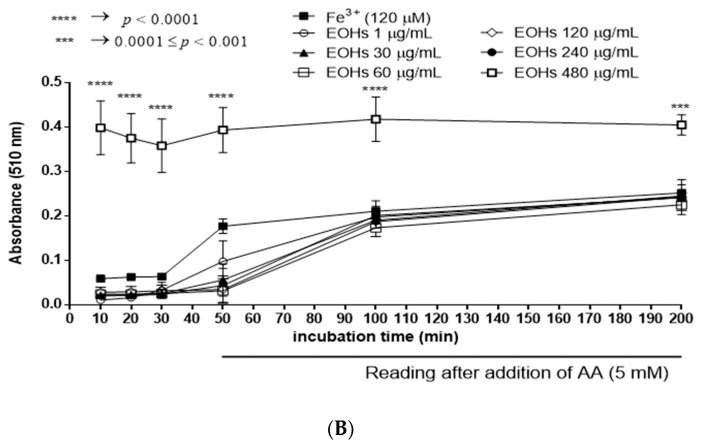
(**A**) Reduction of DPPH radicals by the essential oil of *Rhaphiodon echinus* leaves. Data are expressed as mean ± SEM of *n* = four independent experiments. (**B**) Reduction of Fe^3+^ to Fe^2+^ (110 µM) by the essential oil from *Rhaphiodon echinus* leaf (1–480 µg/mL). The oil was incubated for 10, 20, 30, 50, 100, and 200 min.

In all concentrations (1, 30, 60, 120, 240, and 480 mg/mL) of the essential oil of *Rhaphiodon echinus* evaluated for its antioxidant activity, no relevant effect was observed on the ability to reduce the DPPH radical, so that the IC_50_ of the EORe was 742.80 μg/mL, while the IC_50_ of ascorbic acid, a potent antioxidant, was 41.51 μg/mL, about 18 times lower (Figure 2A).

The power to reduce Fe^3+^ to Fe^2+^ of the essential oil of *R. echinus* (Figure 2B) was similar in almost all concentrations (1, 30, 60, 120, and 240 μg/mL) in the respective checking times (10, 20, 30, 50, 100, and 200), presenting lower absorbance values than those observed in the control curve (Fe^3+^). Only at the concentration of 480 μg/mL did the essential oil demonstrate high reducing power at all times analyzed. After the addition of ascorbic acid (AA), the absorbance values increased in all groups, which is due to the high antioxidant power of this substance.

### 2.4. Cytotoxic Activity of R. echinus against Mammalian Fibroblasts

**Figure 3:**
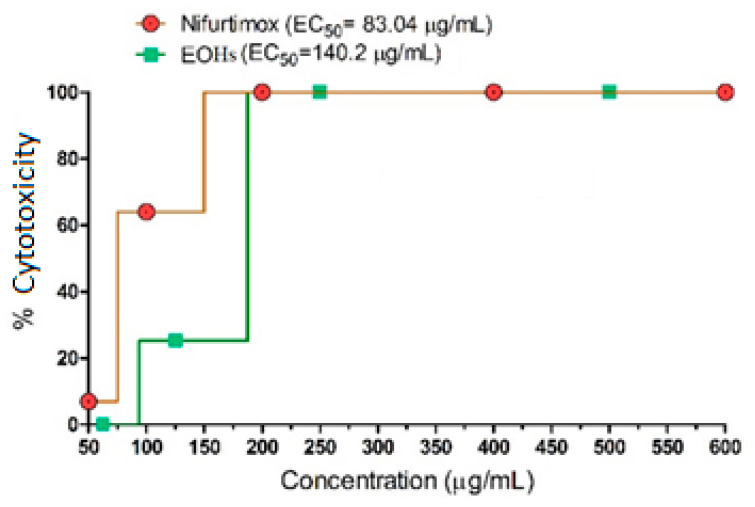
Cytotoxicity of the essential oil of *Rhaphiodon echinus*. As for the absence of error bars, the software used does not efficiently present such graphic elements since they have a negligible value [16].

The cytotoxic potential of the essential oil from the leaves of *R. echinus* against NCTC929 fibroblasts is shown in Figure 3. The essential oil from *R. echinus* at concentration of 250 μg/mL completely killed fibroblasts, while the same effect was obtained with nifurtimox (used as reference) at concentrations ranging from 200 to 600 μg/mL (data not shown). The order of effectiveness to kill the fibroblast was: nifurtimox (EC_50_ = 83.04 μg/mL) > essential oil of *R. echinus* (EC_50_ = 140.2 μg/mL) (Figure 3).

### 2.5. Bioactivity of EOHs for Promastigotes Forms of Leishmania brasiliensis and Epimastigotes of Trypanosoma cruzi

**Figure 4:**
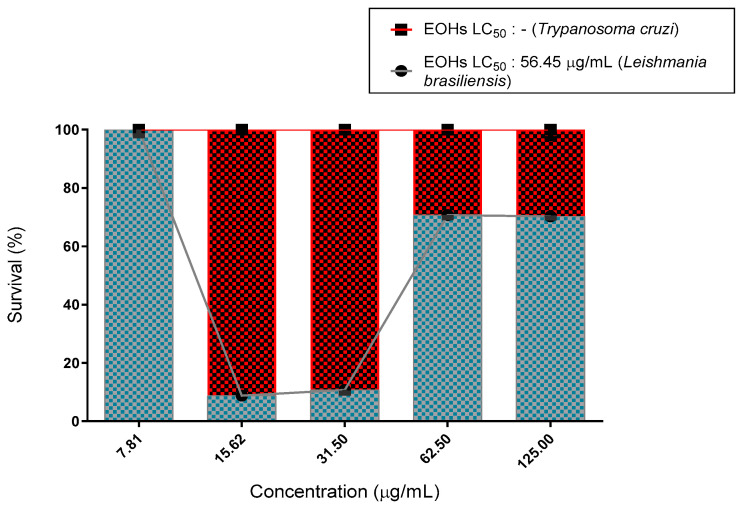
Survival of *Leishmania brasiliensis* promastigotes and *Trypanosoma cruzi* epimastigotes treated with essential oil of *Rhaphiodon echinus*, and their respective LC_50_ values.

To investigate the leishmanicidal activity, promastigotes of *L. brasiliensis* were incubated in the presence of increasing concentrations of essential oil and cell viability was determined 48 h later. As shown in Table 2 and Figure 4, both parasite growths were inhibited by the essential oil. The calculations of LC_50_ values obtained were 56.45 μg/mL and 5.72 μg/mL of essential oil and pentamidine, respectively. 

Our results indicate that the essential oil of *R. echinus* was less effective against the epimastigote forms of *T. cruzi* than against the promastigote forms of *L. brasiliensis* (Figure 4), so that LC50 was observed only for the values of nifurtimox (3.04 μg/mL) at the concentrations tested (Table 2) in the anti-epimastigote assay.

The essential oil of *R. echinus* did not inhibit the growth of *T. cruzi* in any of the tested concentrations, when compared to the control. However, pentamidine was more effective as an anti-kinetoplastida agent than the essential oil of *R. echinus*, since the concentration required to kill 50% (LC50) of parasites was 5.72 μg/mL, while the calculation of LC50 for the essential oil of *R. echinus* was 56.45 μg/mL, in the case of *L. brasiliensis*.

## 3. Discussion

### 3.1. Chemical Composition Analysis of the Essential Oil of R. echinus

In the essential oil of *R. echinus*, various substances are described (Table 1). These natural compounds found are responsible for the anti-inflammatory and antimicrobial actions [17]. Torres et al. [18] evaluated the chemical compositions of the essential oil from leaves and fruits of *R. echinus* by GC–MS and GC–FID and identified nineteen compounds, 93.8% in the leaf oil and 82.4% in the fruit oil. Both presented sesquiterpenes, of which (31.9% and 19.7%) bicyclogermacrene and (21.5% and 21.2%) trans-caryophyllene. *Hyptis sideritis* oil has a terpenic nature, containing monoterpenes and sesquiterpenes, similar to what was observed in *Hyptis suaveolens* (L.) Poiteau, whose essential oil was significantly toxic, with the difference that, in this last one, the main component identified was the oxygenated sesquiterpene β-caryophyllene [19].

In the present study, the major component identified (21.83%) was the sesquiterpene γ-elemene commonly assessed as larvicide [20]. The second most abundant compound in the essential oil of *R. echinus* (10.82%) was α-bisabolene, which is commonly used as a perfume component and as a precursor in several chemical synthesis pathways. In addition to these more common applications, bisabolene has also been shown to be suitable for the synthesis of biofuels for both land and air transport [21].

### 3.2. Visualization Analysis of Descriptor Networks

By analyzing the correlation network obtained using some terms of greater relevance to the present work (Figure 1), such as the compounds identified in the essential oil of *R. echinus*, few works have been carried out with the essential oil of this species, since none of the compounds identified here have been cited as keywords next to the name of this plant, in different research studies. In addition, we noticed that the compounds aromadendrene, caryophyllene oxide, cineole, copaene, germacrene D, spathulenol, α-humulene, and β-pinene seem to be closely correlated, since they were already mentioned together (at least one pair at a time) in at least one job; this seems to be common [22,23].

### 3.3. Effects of the Essential Oil on DPPH Radicals

The results observed for antioxidant activity (Figure 2A) contrasted with those observed for the aqueous and ethanolic extracts of *R. echinus*, where the IC50 values in the DPPH radical reduction assay were 227.9 and 112.9 μg/mL, respectively. Differently, the essential oil of *R. echinus* also showed reducing power, supposedly inferior to the control (Fe^3+^) in lower concentrations, corroborating the findings of the present study [13]. Similarly, it was reported that species of the *Hyptis* genus, such as *H. suaveolens*, produced essential oil with good antioxidant activity [24]. In the case of reducing power, the results (Figure 2B) are similar to those of the work by de Oliveira et al. [25], where the reducing potential of the essential oil of *Lantana montevidensis* was quite high at the highest concentration tested (0.48 g/mL). In the work by Duarte et al. [13], the essential oil of *R. echinus* also showed reducing power supposedly inferior to the control (Fe^3+^) in lower conceptions, corroborating the findings of the present study.

### 3.4. Cytotoxic Activity of R. echinus against Mammalian Fibroblasts

In this study, the cytotoxicity of *R. echinus*, using mammalian fibroblasts, was assessed; the calculation of EC_50_ for the cytotoxic activity was 140.2 µg/mL, showing a slightly different profile from nifurtimox (Figure 3), the standard drug with EC_50_ = 83.04 µg/mL, revealing that the essential oil tested was less active regarding cytotoxicity. 

In similar tests with the same cell line, observed in the literature, concentrations <100% demonstrated similar behavior and were generally null [26,27]; therefore, we used higher concentrations, so that we visualized well-defined behavior characteristics of a dose-dependent response [28].

In studies by Monzote et al. [29], with EC_50_ values between 12.8 and 63.3 μg/mL, the results were similar to those exhibited by the commonly used drugs. The main negative aspects of therapies for leishmaniasis and Chagas disease include high toxicity and high rates of development of resistance by the parasites. The resistance has been observed in vitro [30,31], and this may be associated with a decrease in mitochondrial membrane potential with reduced drug accumulation in prolonged therapies [32]. 

In eukaryotic cells, essential oils can cause depolarization of mitochondrial membranes by decreasing membrane potential, affecting the Ca^2+^ ionic cycle and other ion channels and reducing the pH gradient, affecting the proton pump and the fusion of ATP (adenosine triphosphate) [33]. These cytotoxic properties are of great importance in the application of essential oils, not only against certain human or animal pathogens, but also in the preservation of agricultural products, including the control of mites [34].

It is believed that the presence of secondary metabolites is related to the plant defense [35]. For example, secondary products involved in plant defense through cytotoxicity to pathogens may be useful as antimicrobial drugs in humans if they are not too toxic [36] and can provide valuable information for the screening of natural products [37]. The toxicity involves the formation of pores along the artificial cell membrane of the parasite and host by modifying the selective permeability to cations, leading to cell death [38]. Glinma et al. [39] reported usage of thiosemicarbazones, such as N (4) and N-methyl (4) phenyl, showing antitrypanosomal activity proportional to its lipophilicity. 

### 3.5. Bioactivity of EOHs for Promastigotes Forms of Leishmania brasiliensis and Epimastigotes of Trypanosoma cruzi

Comparing our results with those obtained by Barros et al. [40], using essential oil of *Lantana camara*, it is possible to extrapolate that essential oil *R. echinus* used in this study (Figure 4 and Table 2) was slightly more effective against *L. braziliensis* (LC_50_ = 56.45 μg/mL) than *L. camara* (LC_50_ = 72.31 μg/mL). Senhilkumar et al. [41] found that the essential oil, containing γ-elemene as one of the major constituents, showed significant toxicity, with LC_50_ = 71.71 ppm and LC_90_ = 143.41 ppm. 

The levels of monoterpenes and sesquiterpenes with reports of identified antiparasitic activity may have been responsible for the bioactivity of the oil against *L. brasiliensis*, as the leishmanicidal activity of these terpenoids has already been demonstrated [42,43]. Another component of the essential oil of *R. echinus* is the sesquiterpene caryophyllene oxide, which the literature reports as a significant antitripomastigote activity [44]. For Rondon et al. [45], the more effective action of oils may be due to the synergistic effect of other compounds, such as caryophyllene and cymene, against *Leishmania*.

The probable cause of the change in the response to exposure to EORe, observed here at concentrations greater than 31.5 μg/mL for *L. brasiliensis*, was the change in the cellular behavior of the promastigote forms, so that these must have changed their cellular physiology towards the amastigote condition, a form of resistance, probably due to an increase in the production of reactive oxygen species (ROS) in the intracellular environment [46].

Recent studies have demonstrated the activity of the essential oil component containing caryophyllene [47] and trans-caryophyllene against *T. cruzi*. The effect observed in this study is likely related to a possible synergistic action of the constituents, for how much the various compounds present in the essential oil of *R. echinus* occur show antiparasitic action. Essential oils of aromatic plants showed activity against promastigote (MIC 0.0097–0.1565 μL/mL) and axenic amastigote forms (LC_50_ 0.24–42.00 μL/mL) of both leishmania species [48].

Morais-Braga et al. [37], evaluating the anti-kinetoplastida activity of *Lygodium venustum*, found that promastigotes were more susceptible to the tested products than epimastigotes due to variability and specificity of cellular targets for each organism. In this study, the promastigotes were more susceptible to the tested product. For Nakamura et al. [49], an important criterion in the research of active compounds with therapeutic potential against *L. amazonensis* was to determine the absence of toxic effects on host cells. Comparing the concentration 62.5 μg/mL of greater inhibition with 70.81% of activity for *R. echinus*, with cytotoxicity in this same concentration, there was a total absence of toxic effects—they were not toxic to fibroblasts.

The results demonstrated that *R. echinus* showed no inhibition against *T. cruzi* (125 μg/mL). The toxicity of the essential oil of *R. echinus* is possibly related to the presence of sesquiterpenes. In line with this, Martínez-Diaz et al. [50] demonstrated that (E)-caryophyllene exhibited antiparasitic effects against *T. cruzi*. Similarly, Cheikh-Ali et al. [51] observed the activity of caryophyllene oxide against *T. brucei*. 

Literature reports indicate that essential oils of various plants have shown promising antiparasitic activity against *T. cruzi* [37,52,53]. Saeidnia et al. [54] state that, among various compounds, sesquiterpene lactones showed potent antitrypanosomal effects with a proper selectivity index comparable to trypanocidal drugs. The authors also report that there is no report on the classification of active sesquiterpenes in relation to their activity against several intermediary trypanosomes, such as epimastigotes, trypomastigotes, and amastigotes. 

## 4. Materials and Methods

### 4.1. Plant Material

*R. echinus* was collected in Crato, Ceará, Brazil. The plant material was deposited in the Cariense Dárdano de Andrade-Lima Herbarium of the Regional University of Cariri URCA under the number 7348 HCDAL. 

### 4.2. Reagents

The sodium resazurin substance was obtained from Sigma-Aldrich (St. Louis, MO, USA) and stored at 4 °C protected from light. A resazurin solution was prepared with 1% phosphate buffer, pH 7 and was sterilized in advance by filtration. Afterwards, the chlorophenol red-β-D-galactopyranoside (CPRG, Roche, Indianapolis, IN, USA) was dissolved in a solution of Triton X-100 0.9% (pH 7.4). Penicillin G (Ern, SA, Barcelona, Spain), streptomycin (Reig Jofre SA, Barcelona, Spain) and dimethyl sulfoxide (DMSO) were also used.

### 4.3. Preparation of the Essential Oil of R. echinus (EORe)

The essential oil of *R. echinus* was extracted from dried plant material subjected to hydrodistillation in the Clevenger apparatus. After the sampling, the leaves were placed to dry in the sun, crushed into small pieces, and then introduced into a volumetric flask of 1 L, where 300 mL of distilled water was added. The flask was attached to the Clevenger apparatus on a heating mantle and temperature adjustment was carried out until the water boiling point. After boiling, the count time (the 2 h extraction cycle) began. After each extraction cycle, the oil contained in the apparatus was collected with a pipette and stored in amber bottles and then refrigerated. After the extraction, sodium sulfate was used for removal of the aqueous phase present in the essential oil.

### 4.4. Chemical Composition Analysis of the EORe

The chemical composition of the essential oil of *R. echinus* was performed by gas chromatography coupled to mass spectrometry (GC/MS) using a Shimadzu equipment, QP2010 Series. The capillary column used was of the type Rtx-5MS measuring 30 mm long by 0.25 mm of diameter and 0.25 mm of film thickness. Helium was used as carrier gas at a rate of 1.5 μg/mL/min. The injector temperature was 250 °C and in the detector was 290 °C. The column temperature ranged from 60 to 180 °C increasing 5 °C/min and subsequently varied from 180 to 280 °C rising 10 °C/min. The essential oil was diluted in the proportion 1:200 in chloroform with 1 μL being injected. The mass spectrometer was set for an ionization energy of 70 V. The identification of individual components was based on their fragmentation of spectral mass according to their NIST Mass 08 spectral library, retention rates and comparison with published data [55].

### 4.5. Visualization Analysis of Descriptor Networks

As a recovery strategy, the descriptors “Rhaphiodon echinus” and “R. echinus” were searched for in the search fields on the Scopus web (Elsevier). In this perspective, a universe of 32 documents was found between 2009 and February 2021. From these, the results of keywords cited in the same works were extracted from Scopus (32 documents) and analyzed in VOSviewer. VOSviewer is a useful software for bibliometric and scientometric study, in this context, it allows the creation of visualization networks based on metadata [56]. In this case, the similarity visualization (VOS) method was used to analyze the co-occurrence of descriptors [57].

### 4.6. Cell Lines Used

Strains of CL-B5 (clone CL-B5) were used for in vitro evaluation of the activity on *T. cruzi*. Parasites transfected with the β-galactosidase gene of *Escherichia coli* (LacZ) were provided by Dr. F. Buckner through the Gorgas Memorial Institute (Panama). Epimastigote forms cultured in the LIT infusion tryptose liver at 28 °C plus 10% fetal bovine serum (FBS), penicillin 10 U/mL, and 10 μg/mL streptomycin at pH 7.2, were incubated with different concentrations of essential oil (125, 62.5, 31.25, and 15.62 μg/mL) and harvested during the exponential growth phase.

Antileishmanial in vitro activity was determined using promastigotes of *L. braziliensis* (MHOM/CW/88/UA301) at 26 °C, grown in Schneider’s medium for insects, supplemented with 10% (*v*/*v*) fetal calf serum, heat-inactivated, 2% normal human urine (*v*/*v*), plus penicillin and streptomycin. The forms were seeded and incubated with different concentrations of essential oil (125, 62.5, 31.25, and 15.62 μg/mL).

For the cytotoxic activity, the mammalian fibroblast strain NCTC clone 929 was used. The cells were dissolved in DMSO and diluted in RPMI 1640 medium (Sigma) supplemented with 10% fetal bovine serum (FBS) inactivated by heat (30 min at 56 °C), penicillin G (100 U/mL), and streptomycin (100 μg/mL). The cells in the pre-confluence phase were harvested with trypsin. The cells were maintained at 37 °C on a humidified incubator with 5% CO_2_. Essential oil was added at concentrations of 500, 250, 125, 62.5, 31.25, and 15.62 μg/mL. The antileishmanial activity was tested in concentrations that were not toxic to fibroblasts. Trypanocidal activity and cytotoxicity were tested concomitantly.

### 4.7. Antioxidant Potential of the EORe

#### 4.7.1. Effect of the Essential Oil on DPPH Radicals

The radical scavenging ability of the essential oil of *R. echinus* was performed using the stable free radical DPPH (1,1 diphenyl-2-picrylhydrazyl) as described by Kamdem et al. [58], with some modifications. Briefly, 50 μL of essential oil of *R. echinus* at different concentrations (1–480 μg/mL) were mixed with 100 μL of freshly prepared DPPH solution (0.3 mM in ethanol). Then, the plate was kept in the dark at room temperature for 30 min. The reduction in the DPPH radical was measured by monitoring the decrease of absorption at 517 nm using a microplate reader (SpectraMax, Sunnyvale, CA, USA). Ascorbic acid was used as the standard compound (i.e., positive control). The DPPH radical scavenging capacity was measured using the following equation:

% inhibition = 100 − (Asample − Ablank)/Acontrol × 100

where Asample is the absorbance of the tested sample with DPPH; Ablank, the absorbance of the test tube without adding the DPPH, and Acontrol is the absorbance of the DPPH solution. 

#### 4.7.2. Fe^3+^ Reducing Power of *R. echinus* Essential Oil

The Fe^3+^ reducing property of the essential oil was determined using a modified method of Kamdem et al. [59]. A reaction mixture containing saline solution (58 μL, 0.9%, *w*/*v*), Tris-HCl (45 μL, 0.1 M, pH, 7.5), the oil (27 μL, 1–480 μg/mL), and 110 μM FeCl_3_ (36 μL) was incubated for 10 min at 37 °C. Subsequently, 1,10-phenanthroline (34 μL, 0.25%, *w*/*v*) was added and the absorbance of the orange complex formed was measured at 10, 20, 30, 50, 100, and 200 min at 510 nm (against blank solutions of the samples) using the microplate reader SpectraMax (Molecular Devices, Orleans Drive Sunnyvale CA, USA). The same procedure was performed for the control (i.e., Fe^3+^), but without the oil. The absorbance was also determined 20, 70, and 170 min after adding ascorbic acid. This was necessary because the components of the mixture after long periods may oxidize Fe^2+^ to Fe^3+^, leading to a decrease in absorbance that is not related to the reduction of Fe^3+^ to Fe^2+^.

### 4.8. Anti-Epimastigote Assay of Trypanosoma cruzi

The assays were performed according to the procedures described by Vega et al. [60], with crops that have not reached the stationary phase. Epimastigote forms were seeded at 1 × 10^5^ per mL in 200 μL, in 96-well microdilution plates, which were incubated at 28 °C for 72 h. Then, 50 μL of CPRG solution was added to give a final concentration of 200 μM. The plates were incubated at 37 °C for an additional 6 h. The absorbance reading was performed in a spectrophotometer at 595 nm. Nifurtimox was used as reference drug. The concentrations were tested in triplicate. Each experiment was performed twice, separately. The inhibition percentage (% AE) was calculated as follows:

% AE = [(AE_AEB)/(AC_ACB)] × 100

where AE = absorbance of experimental group; AEB = white of compounds; AC = control group of absorbance; CBA = white of culture environment. The essential oil was previously dissolved in DMSO. The concentration of dimethyl sulfoxide (DMSO) used to enable oil solubility was not greater than 0.01%.

### 4.9. Anti-Promastigote Assay of Leishmania brasiliensis 

The assays were performed according to the procedures described by Mikus and Steverding [61], with some adjustments. The activity of the oil was performed in triplicate. Promastigote forms (2.5 × 10^5^ parasites/well) were cultured in 96-well plastic plates. The samples were dissolved in dimethylsulfoxide (DMSO). Different dilutions of the compounds up to 200 mL of the final volume were added. After 48 h at 26 °C, 20 µL of resazurin solution was added and the oxidation reduction was measured at 570 to 595 nm. In each assay, pentamidine was used as the control reference drug. The anti-promastigotes percentages (AP%) were calculated.

### 4.10. Cytotoxicity Assay

A colorimetric assay with resazurin was used to quantify the cell viability, according to Rolón et al. [62]. NCTC 929 fibroblasts were seeded (5 × 10^4^ cells/well) in flat-bottom 96-well microdilution plates of 100 μL, RPMI 1640 medium, for 24 h at 37 °C in 5% CO_2_, for the cells to adhere to the plates. The medium was replaced by different concentrations of drugs in 200 μL of medium and incubated for another 24 h. Growth controls were included. Then, a volume of 20 μL of 2 mM of resazurin solution was added and the plates were placed in the incubator for another 3 h to assess cell viability. The reduction of resazurin was determined by measuring wavelength absorbance at 490 and 595 nm. During the tests, controls with medium and drugs were used. Each concentration was tested three times. The cytotoxicity of each compound was estimated by calculating the percentage of cytotoxicity (% C). 

### 4.11. Statistical Analysis

All assays were performed in triplicate. The results were expressed as the parasite growth inhibitory concentration (IC_50_) and mean ± standard deviation (sd). The analysis was performed using GraphPad Prism software (version 6.0). Values were expressed as mean ± standard error of the mean (SEM). Two-way ANOVA followed by Dunnett’s multiple comparison test were used when appropriate to assess differences between groups and control. The results are considered statistically significant at *p* < 0.05. The IC_50_ values were estimated through non-linear regression.

## 5. Conclusions

The most abundant compounds identified in the essential oil of *R. echinus*, via a correlation network analysis, seem to be closely correlated. The essential oil of *R. echinus* showed antiparasitic activity against *L. brasiliensis* and no activity against *T. cruzi*. The concentration with the best effect had low cytotoxicity and a high antioxidant capacity, demonstrated by the reducing agent, requiring complementary and additional research to allow clinical use. The species can be an important source in the search for new and selective agents for the treatment of tropical diseases caused by protozoa of the genus *Leishmania*. This study, therefore, demonstrates the potential use of the essential oil of *R. echinus* as a source of new agents for the treatment of leishmaniasis.

## Figures and Tables

**Table 1:** Chemical compositions of the essential oil of *Rhaphiodon echinus*.

Components	RT (min) ^a^	(%)
β-cymene	3.16	0.62
β-pinene	3.60	0.99
Cineol	4.17	0.67
Germacrene B	8.18	1.32
Copaene	8.75	1.64
Aromadendrene	8.92	4.81
α-bisabolene	9.40	12.82
α-selinene	9.56	0.72
α-Cubebene	9.77	1.27
α-humulene	9.85	1.23
germacrene D	10.20	10.31
γ-elemene	10.40	21.83
δ-guaiene	10.48	1.17
Cadina-1.4-diene	10.81	1.52
Spathulenol	11.47	8.84
Caryophyllene oxide	11.58	10.61
Viridiflorol	11.67	1.35
δ-cadinol	12.20	3.37
Oxide α-bisabolol B	12.36	2.03
Globulol	12.52	2.72
α-Bisabolol	12.62	3.76
TOTAL	-	100

Relative proportions of the essential oil constituents are expressed as percentages; ^a^ retention indices from the literature (Adams, 1995).

**Table 2:** Antiparasitic activity of the essential oil *Rhaphiodon echinus*.

Form	Conc. µg/mL	% AA	±% SD
Promastigote of *L. brasiliensis*	125	70.27	0.57
	62.5	70.81	0.81
	31.5	10.73	1.08
	15.62 7.81	8.81 0	1.38 2.10
*R. echinus*: LC_50_ (µg/mL)		56.45	0.82
Pentamidine: LC_50_ (µg/mL)		5.72	0.41
Epimastigote of *T. cruzi*	125	0	3.06
	15.62	0	0.58
*R. echinus*: LC_50_ (µg/mL)		-	-
Nifurtimox: LC_50_ (µg/mL)		3.04	0.77

% AA—percentage of promastigotes of *Leishmania braziliensis* killed by pentamidine or essential oil of *Rhaphiodon echinus* and percentage of epimastigotes of *Trypanosoma cruzi* killed after treatment with nifurtimox or *Rhaphiodon echinus* essential oil. ±% SD—standard deviation. Results are the mean of n = three independent experiments performed in triplicate.
